# Application of biological age assessment of Chinese population in potential anti-ageing technology

**DOI:** 10.1186/s12979-018-0140-9

**Published:** 2018-12-18

**Authors:** Xufeng Li, Jiren Zhang, Chen Sun, Yuanyuan Zhang, Rui Cai, Shilin Fu, Jingfen Zheng, Dehai Huang

**Affiliations:** 1 Institute of Economics, School of Social Sciences, Tsinghua University, No.30 Shuangqing Road, Beijing, 100000 People’s Republic of China; 2 Guangdong Institute of Target Tumor Intervention and Prevention, No.1 Lions Lake Road, Qingyuan, 511500 People’s Republic of China

**Keywords:** Ageing biomarkers, Biological age, Chronological age, Double filtration plasmapheresis

## Abstract

**Background:**

This study aimed to construct a biological age assessment formula for the Chinese population and to explore the effectiveness of double filtration plasmapheresis for anti-ageing and longevity.

**Methods:**

915 subjects were recruited, including 584 (63.8%) males and 331 females (36.2%). Male age was 50.94±10.60 (mean±SD), and female age was 51.20±11.84 (mean±SD). 34 blood markers were detected in the laboratory. The ageing biomarkers were determined by statistical correlation analysis and redundancy analysis, and the biological age assessment formula was established by multiple linear regression analysis. Paired sample T test was used to analyse the elimination effect of double filtration plasmapheresis on aging biomarkers.

**Results:**

Based on the comprehensive blood test and analysis, the ageing biomarkers were screened, and the male and female biological age assessment formulas were established. Then, the elimination of ageing biomarkers by double filtration plasmapheresis was examined. Double filtration plasmapheresis can eliminate ageing biomarkers, with an average of 4.47 years decrease in age for males and 8.36 years for females.

**Conclusion:**

So, biological age provides a scientific tool for assessing ageing, and double filtration plasmapheresis is safe and might be effective for anti-ageing and longevity. However, the effect of plasmapheresis is expected to be transient, so further studies are needed to plan the number and range of the plasmapheresis procedures necessary to consistently lower the parameters under study.

## Background

Population ageing is called demographic transition in demography, referring to the process of transformation from young population with high birth rate and high mortality to elderly population with low birth rate and low mortality, leading to an increase in elderly population, that is, social ageing. The ageing of population is a major social issue around the world since the twentieth century. It is also a global and strategic issue in China’s social and economic development. We should adopt an active ageing approach to reach healthy ageing. Healthy ageing means absence of diseases and disabilities, maintenance of high levels of physical and cognitive abilities and preservation of the social and productive activities [1, 2].

The development of population ageing not only affects social, economic and family, but also has a significant impact on the medical and health field. On April 12, 2008, China Insurance Regulatory Commission issued the China Ageing Social Health Risk Management Report, which showed that the prevalence of chronic diseases among the elderly is 60–70%. The prevalence rate is 3.2 times of the whole group, and the disability rate is 3.6 times. If healthy ageing is not adopted to cope with the ageing of population of the whole country, the social and economic development of China will be challenged. Therefore, to deal with the acceleration of national ageing, healthy ageing is the only right decision that is to comprehensively improve the health of the entire elderly population, reduce the prevalence of chronic diseases and the rate of morbidity, delay ageing and extend healthy life expectancy.

To achieve healthy ageing, we must translate related research achievements into practical applications via further basic and clinical research on ageing. Ageing can be defined as the degenerative process of the biological system [3], the irreversible accumulation of the body’s degenerative changes and increased vulnerability to disease. Ageing is not a disease, but it can reduce the threshold of age-related diseases and increase their prevalence. Ageing is cumulative, universal, progressive, intrinsic, and harmful. With the deepening of research on ageing, the main features of ageing are described as [4]: genomic instability, telomere attrition, epigenetic alterations, loss of proteostasis, deregulated nutrient sensing, mitochondrial dysfunction, cellular senescence, stem cell exhaustion, and altered intercellular communication.

Ageing is a highly personalized and complex process [5, 6]. Because the speed of tissue ageing is different, the difference between human individuals increases with age, and “Chronological Age” does not reflect the process of ageing accurately [7]. In terms of medicine and prevention, researchers are concerned with the changes of individuals, with the aim of screening high-risk individuals for ageing and intervening promptly. In order to accurately and scientifically assess ageing, the researchers proposed “Biological Age” (BA) [8], the biological marker or parameter that can assess an individual functional status based on the functional status of their peers of the same chronological age [9].

The study of biological age helps to identify individuals at risk for age-related dysfunction and is the premise and basis for quantitative analysis of biological ageing of individuals. Chinese scholars have also studied the biological age assessment of Chinese population [10–13], but the measurement of ageing biomarkers selected by those studies is cumbersome. Some markers require specialized medical instrument detection and medical expert interpretation, which is not suitable for practical application. Therefore, convenient and effective biological age assessment method should be the focus. In addition, the scientific assessment of ageing is the beginning of solving the problem of ageing. It is important to implement anti-ageing interventions for individuals [14]. However, less attention has been paid to anti-ageing technology at present.

In this study, through cross-sectional and population-based study, with large sample, on the basis of comprehensive detection of 34 blood markers, the biomarkers of ageing in healthy Chinese population were screened out, and the individualized biological age assessment formula of ageing was constructed to quantitatively analyse the law of ageing in the Chinese healthy population. On this basis, the role of double filtration plasmapheresis technology in eliminating ageing biomarkers [see below, Methods] reducing biological age, anti-ageing and longevity is discussed.

The present study aims to provide an important theoretical basis for the individualized evaluation of ageing in China, and to actively explore a potential safe and effective anti-ageing and life extension technology.

## Methods

### Subjects

Of the 3896 self-evaluated healthy people who were examined at the Lion Lake Hospital affiliated to Guangdong Institute of Target Tumor Intervention and Prevention from January 2015 to May 2018, 915 were recruited, including 584 (63.8%) males and 331 females (36.2%). Male age was 50.94±10.60 (mean±SD), and female age was 51.20±11.84 (mean±SD). There was no statistical difference between male and female age. Inclusion criteria: i) blood and urine routine test, blood biochemistry, electrocardiogram and chest radiograph normal; ii) physical examination: BP < 140/90 mmHg, no disturbance in consciousness and psychology, no obvious abnormality in head and neck, chest, abdomen, limbs and nervous system. Exclusion criteria were: patients with a history of chronic diseases; patients participating in other clinical trials.

This study was approved by the Lion Lake Hospital affiliated to Guangdong Institute of Target Tumor Intervention and Prevention Ethics Committee, and all participants signed informed consent. This study is a cross-sectional study with a combination of epidemiological field investigations, clinical examinations and laboratory tests. The subjects were healthy adults, and the examination for subjects included epidemiological investigations like previous disease investigations, physical examinations, psychological assessments, living conditions, and so on; blood and urine samples were collected and blood, urine routine, blood biochemistry and laboratory markers were tested according to the inclusion criteria and procedures.

### Laboratory tests

The following 34 laboratory tests were performed, according to routinely procedures, at the Lion Lake Hospital affiliated to Guangdong Institute of Target Tumor Intervention and Prevention Molecular Medicine Center: Alanine aminotransferase (ALT), Aminotransferase aspartate (AST), Glutamyl transpeptidase (GGT), alkaline phosphatase (ALP), total protein (TP), albumin (ALB), globulin (GLB), total bilirubin (TBIL), direct bilirubin (DBIL), indirect bilirubin (IBIL), total bile acid (TBA), cholinesterase (CHE), cholesterol (CHOL), triglyceride (TG), high density lipoprotein cholesterol (HDL), low density lipoprotein cholesterol (LDL), Apolipoprotein A (APOA), apolipoprotein B (APOB), lipoprotein a (LP-a), Immunoglobulin E (IGE), Immunoglobulin G (IGG) Immunoglobulin A (IGA), Immunoglobulin M (IGM), urea (UERA), creatinine (CREA), uric acid (UA), β_2_-microglobulin (β_2_M), Blood glucose (GLU), lactate dehydrogenase (LDH), Creatine Kinase (CK), Creatine Kinase-MB (CKMB), Hydroxybutyrate dehydrogenase (HBDH), homocysteine (HCY), Cystatin C (CYSC).

### Double filtration plasmapheresis intervention technology

Double filtration plasmapheresis (DFPP) is a method of selectively removing macromolecular pathogenic substances by filtering plasma twice using a separator and plasma fractionators. DFPP is an integrated blood purification technology that combines two similar blood purification technologies: plasma separation technology and plasma component separation technology [15]. DFPP was performed by using a plasma separator (Plasmaflow OP-05 W Asahi Kasai) and plasma fractionators (EC-40 W Asahi Kasei, Tokyo, Japan). Nine hundred and fifteen subjects underwent double filtration plasmapheresis to detect relevant ageing biomarkers and assessed the biological age of male and female, respectively. Biomarkers were detected on the same day, after the end of plasmapheresis.

### Statistical analysis

In order to select biomarkers of ageing, firstly, the variables not correlated with chronological age (i.e. *p* ≤ 0.05 and *R* ≤ 0.15) in both males and females were excluded. Secondly, correlation matrixes of gender groups were generated to examine their interrelationships and to eliminate possible redundant variables, especially those variables derived from the same organ or system with a very high correlation coefficient (R>|0.3|). Thirdly, multiple linear regression analysis was applied for the selected variables and chronological age to construct the biological age assessment formula of male and female group. Significance of values differences between males and females was performed by Anova. Paired sample T test was used to analyse the elimination effect of double filtration plasmapheresis on aging biomarkers. Statistical analyses were performed with the program PASW statistics 18.0.

## Results

### The situation of blood laboratory markers between different gender groups

Of the 34 laboratory markers, 24 were significantly different between males and females, i.e.: ALT, AST, GGT, ALB, GLB, TBIL, DBIL, IBIL, CHE, GLU, CHOL, TG, HDL, APOA, APOAB, UERA, CERA, UA, HCY, LDH, CK, CKMB, HBDH. (Table 1).

**Table 1:** Laboratory tests in males and females

Marker	Male (*n* = 584) mean ± SD	Female (*n* = 331) mean ± SD	P
ALT	32.20 ± 18.49	23.91 ± 17.2	< 0.001
AST	21.58 ± 9.16	23.91 ± 17.2	N.S.
ALP	69.55 ± 21.22	69.72 ± 22.87	N.S.
GGT	43.96 ± 46.76	23.63 ± 19.65	< 0.001
TP	69.57 ± 4.65	69.63 ± 4.77	N.S.
ALB	45.87 ± 3.20	44.97 ± 3.28	< 0.001
GLB	23.69 ± 3.76	24.66 ± 3.57	< 0.001
TBIL	12.99 ± 5.85	10.72 ± 4.11	< 0.001
DBIL	3.47 ± 1.82	2.64 ± 1.47	< 0.001
IBIL	9.52 ± 4.53	8.11 ± 3.14	< 0.001
TBA	9.22 ± 6.60	8.95 ± 6.61	N.S.
CHE	9272.32 ± 1957.16	8728.16 ± 1961.14	< 0.001
GLU	7.27 ± 3.48	6.61 ± 3.02	=0.003
CHOL	5.05 ± 0.99	5.23 ± 1.05	=0.010
TG	3.04 ± 2.04	2.23 ± 1.83	< 0.001
HDL	1.17 ± 0.29	1.42 ± 0.34	< 0.001
LDL	2.92 ± 0.82	2.96 ± 0.81	N.S.
APOA	1.25 ± 0.22	1.38 ± 0.25	< 0.001
APOB	0.97 ± 0.32	0.95 ± 0.29	N.S.
IGG	11.5 ± 2.57	12.56 ± 2.77	< 0.001
IGA	2.43 ± 1.01	2.30 ± 0.98	N.S.
IGE	139.45 ± 234.56	109.83 ± 179.50	=0.033
IGM	0.98 ± 0.58	1.35 ± 0.78	< 0.001
UERA	5.43 ± 1.34	4.97 ± 1.45	< 0.001
CREA	88.94 ± 20.42	77.25 ± 15.4	< 0.001
UA	410.48 ± 83.70	72.24 ± 15.40	< 0.001
β2M	0.99 ± 0.52	0.99 ± 0.51	N.S.
HCY	12.62 ± 4.61	11.24 ± 4.16	< 0.001
CYSC	0.77 ± 0.28	0.73 ± 0.22	N.S.
LDH	168.63 ± 29.33	177.86 ± 35.19	< 0.001
CK	157.18 ± 186.75	109.08 ± 58.44	< 0.001
CKMB	12.74 ± 4.90	11.71 ± 6.92	=0.009
HBDH	138.87 ± 25.96	150.37 ± 32.08	< 0.001

Significance by ANOVA

*ALB* albumin, *ALP* alkaline phosphatase, *ALT* Alanine aminotransferase, *APOA* Apolipoprotein A, *APOB* apolipoprotein B, *AST* Aminotransferase aspartate, *β*_*2*_*M* β_2_-microglobulin, *CHE* cholinesterase, *CHOL* cholesterol, *CK* Creatine Kinase, *CKMB* Creatine Kinase-MB, *CREA* creatinine, *CYSC* Cystatin C, *DBIL* direct bilirubin, *GGT* Glutamyl transpeptidase, *GLB* globulin, *GLU* Blood glucose, *HBDH* Hydroxybutyrate dehydrogenase, *HCY* homocysteine, *HDL* high density lipoprotein cholesterol, *IBIL* indirect bilirubin, *IGA* Immunoglobulin A, *IGE* Immunoglobulin E, *IGG* Immunoglobulin G, *IGM* Immunoglobulin M, *LDH* lactate dehydrogenase, *LDL* low density lipoprotein cholesterol, *LP-a* lipoprotein-a, *TBA* total bile acid, *TBIL* total bilirubin, *TG* triglyceride, *TP* total protein, *UA* uric acid, *UERA* urea

### Correlation analysis of laboratory markers and chronological age and redundancy analysis between markers

Correlation analysis between markers and chronological age (male): in order to ensure sufficient marker flux and improve the comprehensiveness of the evaluation, this study used (*r* > 0.15, *p* < 0.001) as the inclusion criteria for chronological age-related variables. Relevant analysis of ALT, TP, ALB, CHE, HCY, IGM, CYCS, UERA, CERA, GLU, β_2_M met the requirements (Table 2), which were selected as markers for the redundancy analysis.

**Table 2:** Correlation analysis between chronological age and selected laboratory tests in males

Indicator	Correlation coefficient R	*P* value	Indicator	Correlation coefficient R	*P* value
ALT	-0.239	<0.001	ALP	-0.099	=0.023
AST	-0.146	<0.001	LPA	0.095	=0.022
GGT	-0.127	=0.002	IGM	-0.163	<0.001
TP	-0.223	<0.001	UERA	0.157	<0.001
ALB	-0.327	<0.001	CREA	0.218	<0.001
TBA	0.114	=0.006	GLU	0.202	<0.001
CHE	-0.194	<0.001	UA	-0.112	=0.007
HCY	0.226	<0.001	β_2_M	0.333	<0.001
CYCS	0.260	<0.001	TG	-0.114	=0.006

Pearson correlation analysis

A correlation matrix for each marker was established and redundant variables were excluded, i.e. variables derived from the same organ or system that were highly correlated with each other (*r* > 0.3). The markers of higher Correlation coefficient with chronological age were selected for further analysis. After redundant analysis, ALB, IGM, UERA, β_2_M, GLU, CYCS, HCY were used for the biological age regression analysis (Table 3).

**Table 3:** Correlation matrix between 11 markers in males

	ALT	TP	ALB	IGM	UERA	CERA	β_2_M	GLU	CYCS	HCY	CHE
ALT	1	0.257 <0.001	0.550 <0.001	0.055 0.185	-0.019 0.649	0.009 0.835	-0.050 0.224	0.043 0.296	-0.060 0.152	-0.064 0.124	0.146 <0.001
TP		1	0.595 <0.001	0.339 <0.001	0.008 0.839	0.081 0.051	-0.085 0.040	-0.086 0.038	0.091 0.029	-0.077 0.062	0.272 <0.001
ALB			1	0.070 0.089	-0.134 0.001	0.061 0.143	-0.234 <0.001	-0.149 <0.001	-0.105 0.012	-0.085 0.040	0.364 <0.001
IGM				1	0.031 0.452	0.004 0.982	-0.009 0.821	-0.055 0.181	0.002 0.961	0.034 0.419	-0.019 0.645
UERA					1	0.481 <0.001	0.188 <0.001	0.020 0.632	0.115 0.005	0.016 0.691	0.043 0.296
CERA						1	0.175 <0.001	0.017 0.686	0.089 0.033	0.147 <0.001	0.164 <0.001
β_2_M							1	0.021 0.614	0.221 <0.001	0.140 0.034	-0.196 <0.001
GLU								1	0.017 0.682	0.052 0.206	0.045 0.278
CYCS									1	-0.027 0.523	-0.109 0.008
HCY										1	-0.137 0.001
CHE											1

Pearson correlation analysis

Correlation analysis between markers and chronological age (female): in order to ensure sufficient marker flux and improve the comprehensiveness of the evaluation, this study used (*r* > 0.15, *p* < 0.001) as the inclusion criteria for chronological age-related variables. Correlation analysis showed that ALP, TG, ALB, CHE, CHOL, LDL, β_2_M, IGM, UERA, CERA, GLU, LDH, CKMB, HCY, HBDH met the requirements and were chosen as markers for biological age analysis (Tables 4 and 5).

**Table 4:** Correlation analysis between chronological age and selected laboratory tests in females

Marker	Correlation coefficient R	P value	Marker	Correlation coefficient R	P value
ALP	0.321	<0.001	ALB	-0.170	=0.007
TP	-0.109	=0.048	CHE	0.225	<0.001
TBA	0.143	=0.009	TG	0.280	<0.001
CHOL	0.204	<0.001	LDL	0.192	<0.001
HDL	-0.150	=0.007	UERA	0.396	<0.001
IGM	-0.292	<0.001	β_2_M	0.420	<0.001
CREA	0.233	<0.001	LDH	0.444	<0.001
GLU	0.328	<0.001	CKMB	0.204	<0.001
CK	0.144	=0.009	HCY	0.322	<0.001
HBDH	0.425	<0.001			

Pearson correlation analysis

**Table 5:** Correlation matrix between 11 markers in females

	ALP	ALB	CHE	CHOL	TG	LDL	GIM	UERA	CERA	β_2_M	GLU	LDH	CKMB	HBDH	HCY
ALP	1	0.009 <0.001	0.384 <0.001	0.220 <0.001	0.263 <0.001	0.237 <0.001	0.086 0.118	0.111 0.043	0.207 <0.001	0.123 0.025	0.331 <0.001	0.267 <0.001	0.087 0.114	0.190 <0.001	0.133 0.016
ALB		1	0.165 0.003	0.175 0.001	-0.08 0.147	0.166 0.002	0.072 0.194	-0.143 0.009	0.046 0.400	-0.221 <0.001	-0.095 0.083	0.112 0.041	0.024 0.685	0.017 0.761	-0.027 0.619
CHE			1	0.363 <0.001	0.428 <0.001	0.395 <0.001	0.010 0.859	0.143 0.009	0.243 <0.001	-0.044 0.420	0.289 <0.001	0.282 <0.001	0.001 0.991	0.198 <0.001	0.000 0.999
CHOL				1	0.393 <0.001	0.804 <0.001	-0.026 0.634	0.134 0.015	0.074 0.182	-0.043 0.432	0.031 0.578	0.178 0.001	0.032 0.556	0.169 0.002	-0.001 0.988
TG					1	0.198 <0.001	-0.110 0.045	0.199 <0.001	0.113 0.040	0.128 0.02	0.378 <0.001	0.232 <0.001	0.173 0.002	0.193 <0.001	0.114 0.038
LDL						1	-0.030 0.592	0.151 0.006	0.029 0.596	0.000 0.998	0.024 0.667	0.142 0.010	-0.088 0.109	0.091 0.098	-0.055 0.317
IGM							1	-0.184 0.001	0.049 0.375	-0.116 0.034	-0.019 0.731	-0.150 0.006	-0.124 0.024	-0.015 0.788	-0.063 0.252
UERA								1	0.104 0.060	0.314 <0.001	0.253 <0.001	0.301 <0.001	0.059 0.281	0.336 <0.001	0.211 <0.001
CERA									1	0.052 0.346	0.146 0.006	0.064 0.246	0.186 0.001	0.110 0.045	0.140 0.011
β_2_M										1	0.015 <0.001	0.254 <0.001	0.118 0.032	0.259 <0.001	0.275 <0.001
GLU											1	0.203 <0.001	0.070 0.204	0.125 0.023	0.097 0.078
LDH												1	0.043 0.434	0.851 <0.001	0.241 <0.001
CKMB													1	0.103 0.062	0.072 0.189
HBDH														1	0.070 0.207
HCY															1

Pearson correlation analysis

### Biological age assessment formula, correlation between biological age and chronological age

The numbers of regression equations are derived from multiple linear regression analysis (Tables 6 and 7). The constant terms and coefficients of each index are given in the tables. Because there are gender differences in the markers of aging between males and females, multiple linear regression analysis was performed for males and females respectively in establishing regression equations. So, the regression equations between male and female were different**.**

1. Biological age assessment formula (male)

**Table 6:** Biological Age Regression analysis in males

Model	Coefficient	T	P
Constants	69.204	11.044	<0.001
ALB	-.713	-5.903	.000
IGM	-2.588	-4.070	.000
UERA	.366	3.211	.000
β_2_M	2.992	3.579	.000
HCY	.358	4.475	.000
GLU	.424	3.944	.000
CYSC	6.185	5.438	.000

Biological age assessment (Formula 1). Biological Age = 69.204-2.588×IGM-0.713×ALB+0.366×UERA+2.992×β_2_M+6.185×CYCS+0.358×HCY +0.424×GLU

Multiple linear regression

**Table 7:** Biological Age Regression Analysis in females

Model	Coefficient	T	P
Constants	28.235	3.796	.000
ALP	.074	3.142	.002
ALB	-.445	-2.888	.004
LDL	1.829	2.956	.003
IGM	-3.415	-5.449	.000
UERA	.784	2.116	.035
CERA	.114	3.604	.000
β2M	5.218	5.065	.000
GLU	.365	2.096	.007
LDH	.077	5.068	.000
HCY	.329	2.897	.004

Female biology age assessment formula: BA (female)=28.235+0.074×ALP-0.445×ALB+1.829×LDL-3.415×IGM+0.784×UERA+0.114×CREA+5.218×β_2_M+0.365×GLU+0.077×LDH+0.329×HCY (formula 2)

multiple linear regression

After correlation analysis and redundancy analysis, 7 ageing biomarkers were screened and multiple linear regression analysis was used to construct the biological age assessment formula (Table 6).

Male Biological age assessment (eq. 1).

$$ BA(male)=69.204-2.588\times IGM-0.713\times ALB+0.366\times UERA+2.992\times {\beta}_2M+6.185\times CYCS+0.358\times HCY+0.424\times GLU $$

(2) Correlation between biological age and chronological age (male)

Pearson correlation analysis of male biological age and chronological age showed that correlation coefficient *R* = 0.882, *p* < 0.001, indicating that the correlation between biological age and chronological age was significant.

(3) Biological age assessment formula (female)

After correlation analysis and redundancy analysis, 10 ageing markers were screened and multiple linear regression analysis was used to construct the biological age assessment formula (Table 7).

Female biology age assessment (eq 2).

$$ BA(female)=28.235+0.074\times ALP-0.445\times ALB+1.829\times LDL-3.415\times IGM+0.784\times UERA+0.114\times CREA+5.218\times {\beta}_2M+0.365\times GLU+0.077\times LDH+0.329\times HCY $$

(4) Correlation between biological age and chronological age (female)

Pearson correlation analysis of female biological age and chronological age showed that correlation coefficient *R* = 0.803, *p* < 0.001, indicating that the correlation between biological age and chronological age was significant.

### Analysis of the discharge of ageing biomarkers by double filtration plasmapheresis intervention technique

Paired T test was used for screened ageing biomakers before and after intervention, and 11 pairs of ageing markers changed after intervention, with statistically significant difference (Table 8).

1 $$ BA(male)=69.204-2.588\times IGM-0.713\times ALB+0.366\times UERA+2.992\times {\beta}_2M+6.185\times CYCS+0.358\times HCY+0.424\times GLU $$

**Table 8:** Changes in markers before and after intervention

Indicator	MEAN±SD	T	P
ALP2 – ALP1	-21.36±10.07	-64.166	.000
ALB2 – ALB1	-0.190±0.07	-78.816	.000
LDL2 – LDL1	-1.24±0.497	-75.534	.000
IGM2 – IGM1	-0.06±0.01	-41.432	.000
UERA2 – UERA1	-0.27±0.51	-15.819	.000
CREA2 – CREA1	-9.75±3.64	-38.611	.000
β2M2 - β2M1	-0.15±0.17	-27.327	.000
GLU2 – GLU1	-0.99±0.34	12.938	.000
LDH2 – LDH1	-33.29±25.39	-39.638	.000
HCY2- HCY1	-4.47±2.50	-17.682	.000
CYSC2 - CYSC1	-0.36±0.09	-17.956	.000

Paired sample T test

The ageing biomarkers before and after intervention were brought into the biological age eq 1, and the biological age after intervention was *BA*2 (*male*) = 69.204 − 2.588 × *IGM*2 + 0.366 × *UERA*2 + 2.992 × *β* _ 2 *M*2 + 6.185 × *CYCS*2 + 0.358 × *HCY*2 − 0.713 × *ALB*2 + 0.424 × *GLU*2, pre-interventional biological age *BA*1(*male*) = 69.204 − 2.588 × *IGM*1 + 0.366 × *UERA*1 + 2.992 × *β*_2_*M*1 + 6.185 × *CYCS*1 + 0.358 × *HCY*1 − 0.713 × *ALB*1 + 0.424 × *GLU*1, *BA*2(*male*) − *BA*1(*male*) represented for the declined biological age after intervention, and the biological age after the intervention was calculated to be decreased by 4.47 years. *BA*(*female*) = 28.235 + 0.074 × *ALP* − 0.445 × *ALB* + 1.829 × *LDL* − 3.415 × *IGM* + 0.784 × *UERA* + 0.114 × *CREA* + 5.218 × *β* _ 2 *M* + 0.365 × *GLU* + 0.077 × *LDH* + 0.329 × *HCY*.

The ageing biomarkers before and after intervention were brought into eq 2, and the biological age after intervention was *BA*2(*female*) = 28.235 + 0.074 × *ALP*2 − 0.445 × *ALB*2 + 1.829 × *LDL*2 − 3.415 × *IGM*2 + 0.784 × *UERA*2 + 0.114 × *CREA*2 + 5.218 × *β*_2_*M*2 + 0.365 × *GLU*2 + 0.077 × *LDH*2 + 0.329 × *HCY*2, pre-interventional biological age *BA*1(*female*) = 28.235 + 0.074 × *ALP*1 − 0.445 × *ALB*1 + 1.829 × *LDL*1 − 3.415 × *IGM*1 + 0.784 × *UERA*1 + 0.114 × *CREA*1 + 5.218 × *β*_2_*M*1 + 0.365 × *GLU*1 + 0.077 × *LDH*1 + 0.329 × *HCY*1, *BA*2(*female*) − *BA*1(*female*) represented for the declined biological age after intervention, and the biological age after the intervention was calculated to be decreased by 8.36 years old.

## Discussion

The ageing of the population is a major social issue around the world since the twentieth century. It is also a global and strategic issue in China’s social and economic development. Healthy ageing is the only way to cope with the ageing population. Achieving healthy ageing is inseparable from the scientific assessment of ageing and the development of safe and effective anti-ageing technologies [1, 2].

This study is a large-sample cross-sectional study. Based on the comprehensive blood test and analysis, the ageing biomarkers were screened to establish the male and female biological age assessment formulas. From the perspective of prevention, the assessment of ageing is only the starting point. The purpose of the assessment is to screen out high-risk individuals, implement targeted interventions for high-risk individuals to achieve anti-ageing and longevity, and reduce the possibility of chronic diseases caused by ageing. Therefore, on the basis of assessing ageing, we explored the elimination of ageing biomarkers by double filtration plasmapheresis. The results showed that double filtration plasmapheresis could eliminate ageing biomarkers. The biological ages of males and females was decreased by 4.47 and 8.36 years after intervention. Double filtration plasmapheresis technology is a safe and effective anti-ageing and life extension technology.

Ageing is a cumulative, universal, progressive, endogenous, and harmful life process that begins or accelerates after reproductive maturity. [16]. Cumulative, that is, ageing is not achieved overnight, it is the performance of long-term accumulation of mild or minor changes, which is irreversible; however, in the future of technological advancement, some changes in ageing may become reality. Universal, ageing is a phenomenon that can be expressed by the same species in the same time frame, and almost all living creatures have ageing process. Progressive, ageing is a continuous evolutionary process. Intrinsic, ageing stems from the inherent nature of the organism, but environmental impact is not completely ruled out. Deleterious, the ageing process is generally unfavourable to life; the function is reduced or even lost; the body is more and more susceptible to disease, and ultimately to death. In the process of ageing, the body undergoes a series of changes from macroscopic to microscopic, mainly manifested as ageing at the whole level, tissue and organ level, cell level, and molecular level [17, 18].

Our assessment on ageing is often based on the individual’s chronological age, but ageing is a highly individualized process, and there are significant individual differences in physiological function degradation [5, 6], so the chronological age is only a rough marker of the ageing process. The purpose of ageing research is to screen high-risk individuals and promptly intervene. Therefore, biological age has been proposed instead of chronological age to assess ageing individuals. Biological age assumes that time factors are indirectly related to ageing, and ageing is a process that is not entirely time dependent [7–9].

Since the early 1990s, developed countries such as European countries, America, and Japan have successively implemented a series of longitudinal studies on ageing. Representative examples are longitudinal ageing studies in BLSA [19] and Health ABC [20] in the United States, VLS studies in Canada [21], 7-year longitudinal studies in Japan [22], and ILSA [23] research in Italy. The main concerns of these studies are: individualized evaluation of ageing, identification of reliable and easily measurable ageing biomarkers and how to achieve healthy ageing. Research on ageing biomarkers and ageing assessment in Chinese population has not attracted widespread attention, and domestic scholars use biological age scores to assess ageing. However, the biomarkers screened in those studies are difficult to measure and require specialized medical instruments and professional interpretation, which is not convenient for promotion [10–13].

Biomarkers of ageing are a set of biological markers that can predict the functional status of the body. Whether it can be tested non-invasively or minimally invasively determines whether it can be promoted or not, and blood test markers have such characteristics [24]. In this study, based on 34 blood test markers, ageing biomarkers were screened and determined, and the biological age assessment formula was further suggested.

Assessing ageing is only the beginning of solving the problem of ageing. The anti-ageing intervention program for high-risk individuals is the end point. In clinical treatment, double filtration plasmapheresis has been approved for the treatment of critically ill patients, but its use in disease prevention has not been reported. This study explored the potential application of double filtration plasmapheresis in anti-ageing. Nine hundred and fifteen subjects underwent biological age assessment before and after intervention. The results confirmed that the biological age of males and females decreased by 4.47 years and 8.36 years after intervention. It is suggested that double filtration plasmapheresis technology might have potential application value in anti-ageing.

Of course, there are some limitations in this study: 1. This study is a single-centre study that does not include sufficient representative sample data. 2. It is well known that the lifestyle such as a healthy dietary pattern may deeply affect biological age [25, 26]. Unfortunately, this information was not available, so we do not know the possible interaction between life style, biomarkers and plasmapheresis. 3. Due to funding limitation in this study, other important ageing biomarkers such as telomere length and ageing biomarkers associated with age-related diseases as arachidonic acid/eicosapentaenoic acid ratio associated with polyunsaturated fatty acids metabolism and cardiovascular disease onset and risk were not analysed [27, 28]. 4. More importantly, due to the limitation of funding, no follow-up was performed on the population after intervention.

## Conclusion

Biological age provides a scientific tool for assessing ageing, and double filtration plasmapheresis technology might have potential application value in anti-ageing. However, the effect of plasmapheresis is expected to be transient, so further studies are needed to plan the number and range of the plasmapheresis procedures necessary to consistently lower the parameters under study. Finally, it has been claimed that ageing processes can be quantified in people still young enough for prevention of age-related disease, opening a new door for antiaging therapies [29]. So, future studies should include younger people.

## Abbreviations

ALB: Albumin
ALP: Alkaline phosphatase
ALT: Alanine aminotransferase
APOA: Apolipoprotein A
APOB: Apolipoprotein B
AST: Aminotransferase aspartate
BA: Biologoical ageing
CHE: Cholinesterase
CHOL: Cholesterol
CK: Creatine Kinase
CKMB: Creatine Kinase-MB
CREA: creatinine
CYSC: Cystatin C
DBIL: Direct bilirubin
DFPP: Double filtration plasmapheresis
GGT: Glutamyl transpeptidase
GLB: Globulin
GLU: Blood glucose
HBDH: Hydroxybutyrate dehydrogenase
HCY: Homocysteine
HDL: High density lipoprotein cholesterol
IBIL: Indirect bilirubin
IGA: Immunoglobulin A
IGE: Immunoglobulin E
IGG: Immunoglobulin G
IGM: Immunoglobulin M
LDH: Lactate dehydrogenase
LDL: Low density lipoprotein cholesterol
LP-a: Lipoprotein-a
TBA: Total bile acid
TBIL: Total bilirubin
TG: Triglyceride
TP: Total protein
UA: Uric acid
UERA: Urea
β_2_M: β_2_-microglobulin
